# Supplementary material for: DNMT3A-mediated epigenetic silencing of SOX17 contributes to endothelial cell migration and fibroblast activation in wound healing
Source: PLoS One. 2023 Oct 19;18(10):e0292684. doi: 10.1371/journal.pone.0292684 (PMC10586696; doi:10.1371/journal.pone.0292684)
Supplement: S1 Table — (DOCX) [file pone.0292684.s001.docx]

Table 1. Primer sequences used in this study

The primers used in RT-qPCR assay

| SOX17 | Forward primer | 5’-GTGGACCGCACGGAATTTG-3’ |
| --- | --- | --- |
|  | Reverse primer | 5’-GGAGATTCACACCGGAGTCA-3’ |
| TGF-β | Forward primer | 5’-GTGGACATCAACGGGTTCAC-3’ |
|  | Reverse primer | 5’-GAAGTTGGCATGGTAGCCCT-3’ |

The primers of SOX17 promoter CpG island used in ChIP-PCR or ChIP-qPCR assay

| SOX17 | Forward primer | GTACAATCAGCCCTCCCAGAC |
| --- | --- | --- |
|  | Reverse primer | GCACAGATGTGGCCAATGGAG |

The primers used in DNA methylation analysis

| SOX17 | F | 5’-AAGTAGTTTGGGAGGGTTGATTGT-3’ |
| --- | --- | --- |
|  | R | 5-ATAAACTAAATCACCCACCACTAA-3’ |
|  | S | 5-GGTTGATTGTATTTTGGAAT-3’ |
