# Supplementary material for: DNMT3A-mediated epigenetic silencing of SOX17 contributes to endothelial cell migration and fibroblast activation in wound healing
Source: PLoS One. 2023 Oct 19;18(10):e0292684. doi: 10.1371/journal.pone.0292684 (PMC10586696; doi:10.1371/journal.pone.0292684)
Supplement: S1 File — (DOCX) [file pone.0292684.s002.docx]

Figure 3F: repetition 1


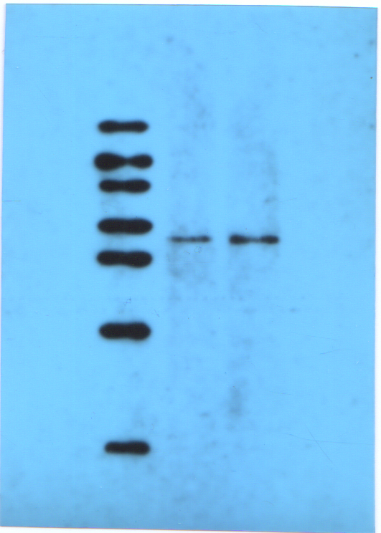

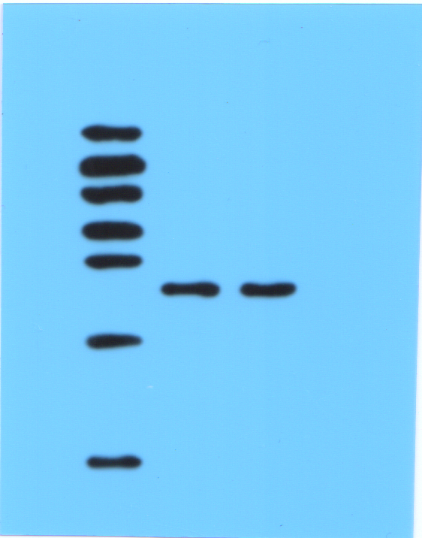


KDa

20

120

80

60

50

40

30

TGF-β GAPDH

Figure 3F: repetition 2


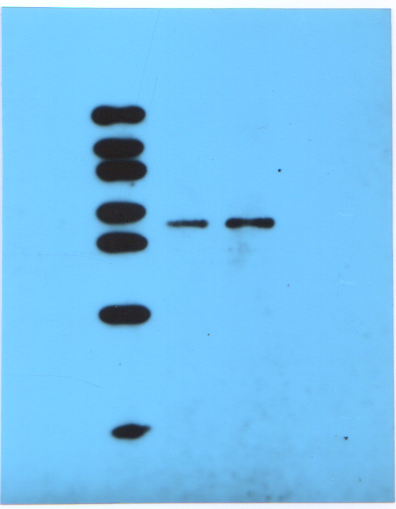

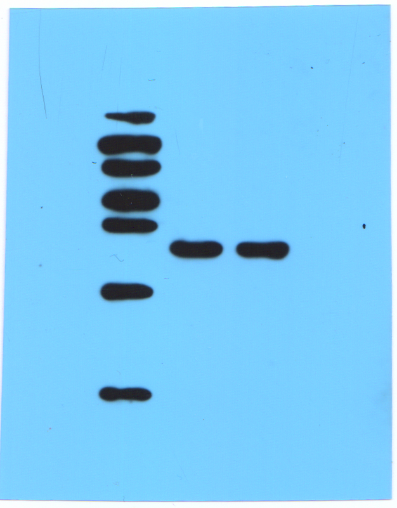


TGF-β GAPDH

Figure 3F: repetition 3


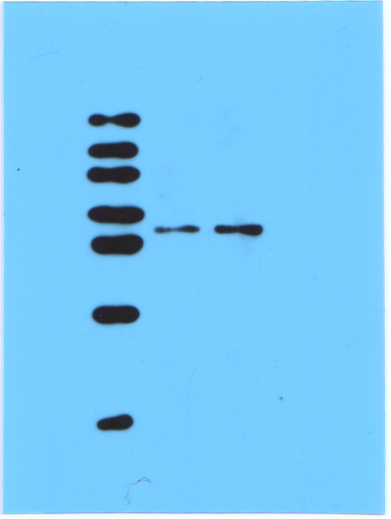

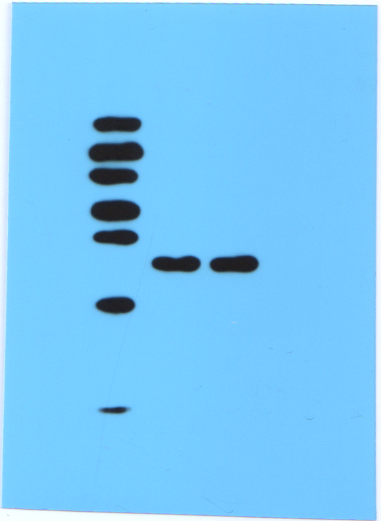


TGF-β GAPDH

Figure 5B


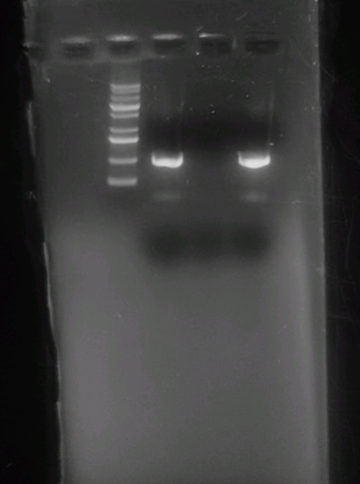


bp

100000

250000

500000

750000

1000000

1500000

2000000

3000000

5000

Figure 5D


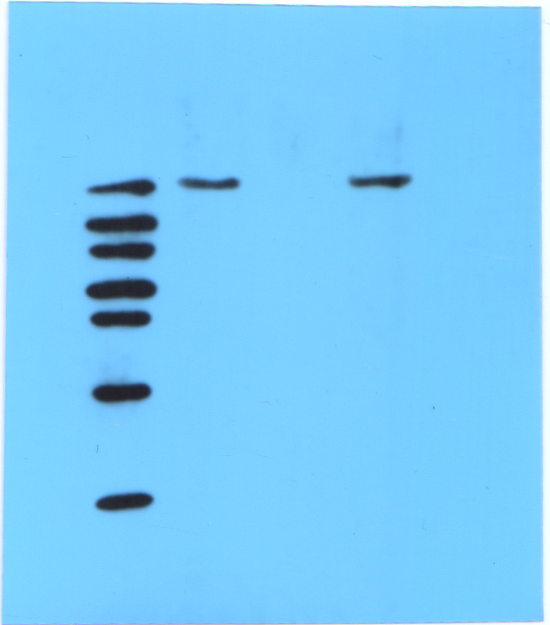


40

50

60

20

30

80

120

KDa

DNMT3A
