# Supplementary material for: DNMT3A-mediated epigenetic silencing of SOX17 contributes to endothelial cell migration and fibroblast activation in wound healing
Source: PLoS One. 2023 Oct 19;18(10):e0292684. doi: 10.1371/journal.pone.0292684 (PMC10586696; doi:10.1371/journal.pone.0292684)
Supplement: S2 File — (DOCX) [file pone.0292684.s003.docx]

DNMT3A-mediated epigenetic silencing of SOX17 promotes wound healing by facilitating the migration of vascular endothelial cells and the activation of fibroblasts.
