# Supplementary figures and images for: DNMT3A-mediated epigenetic silencing of SOX17 contributes to endothelial cell migration and fibroblast activation in wound healing
Source: PLoS One. 2023 Oct 19;18(10):e0292684. doi: 10.1371/journal.pone.0292684 (PMC10586696; doi:10.1371/journal.pone.0292684)

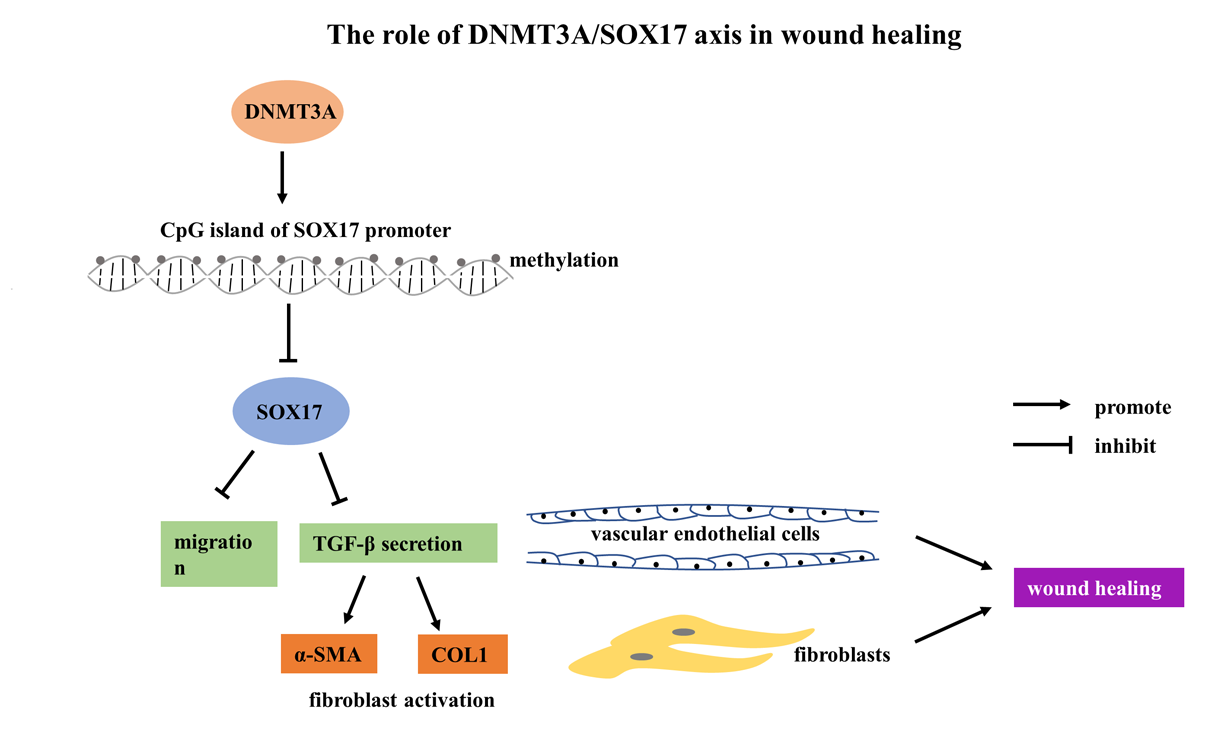

Supplement: S1 Graphical abstract — (TIF) [file pone.0292684.s004.tif]
