# Supplementary material for: DNMT3A-mediated epigenetic silencing of SOX17 contributes to endothelial cell migration and fibroblast activation in wound healing
Source: PLoS One. 2023 Oct 19;18(10):e0292684. doi: 10.1371/journal.pone.0292684 (PMC10586696; doi:10.1371/journal.pone.0292684)
Supplement: Attachment — Submitted filename: Response to Editor.docx [file pone.0292684.s005.docx]

Dear Dr. Zhou,

We've checked your submission and before we can proceed, we need you to address the following issues:

1. PLOS requires an ORCID iD for the corresponding author in Editorial Manager on papers submitted after December 6th, 2016. Please ensure that you have an ORCID iD and that it is validated in Editorial Manager. To do this, go to ‘Update my Information’ (in the upper left-hand corner of the main menu), and click on the Fetch/Validate link next to the ORCID field. This will take you to the ORCID site and allow you to create a new iD or authenticate a pre-existing iD in Editorial Manager. Please see the following video for instructions on linking an ORCID iD to your Editorial Manager account: <https://www.youtube.com/watch?v=_xcclfuvtxQ>

Re: Thank you for your suggestion. We have created an ORCID iD of the corresponding author in Editorial Manager.

2. Please note that funding information should not appear in any section or other areas of your manuscript. We will only publish funding information present in the Funding Statement section of the online submission form. Please remove any funding-related text from the manuscript.

Re: We have deleted the funding-related information in our revised manuscript.

3. Can you please upload an additional copy of your revised manuscript that does not contain any tracked changes or highlighting as your main article file. This will be used in the production process if your manuscript is accepted. Please amend the file type for the file showing your changes to Revised Manuscript w/tracked changes. Please follow this link for more information: http://blogs.PLOS.org/everyone/2011/05/10/how-to-submit-your-revised-manuscript/

Re: We have up-loaded an additional copy of the revised manuscript that does not contain any tracked changes, named “Manuscript”, and the revised manuscript with tracked changes, named “Revised Manuscript with Track Changes”.
